# Supplementary material for: MetaRibo-Seq measures translation in microbiomes
Source: Nat Commun. 2020 Jun 29;11:3268. doi: 10.1038/s41467-020-17081-z (PMC7324362; doi:10.1038/s41467-020-17081-z)
Supplement: Supplementary file 10 — Supplementary Data 7 [file 41467_2020_17081_MOESM10_ESM.zip › File2/Confidence_High_Taxonomy/200965_out.krona.html]

Javascript must be enabled to view this page.

members
magnitude
magnitudeUnassigned
count
unassigned
taxon
rank

200965\_out

11

2
11
superkingdom

976
phylum
11

11
class
200643

11
order
171549

family
11
171552

genus
11
838

2
species
59823

SRS013098\_contig\_number\_14904SRS024132\_contig\_number\_29735

species
2

SRS015590\_contig\_number\_12266SRS100907\_contig\_number\_8038
2292365


SRS019285\_contig\_number\_contig-100\_205.22095SRS043841\_contig\_number\_6265SRS044535\_contig\_number\_10978SRS047433\_contig\_number\_contig-100\_1364.110795SRS078419\_contig\_number\_4815SRS1041137\_contig\_number\_26170SRS150029\_contig\_number\_4783
165179
7
species
